# Psychoactive substance use, internet use and mental health changes during the COVID-19 lockdown in a French population: A study of gender effect

**DOI:** 10.3389/fpsyt.2022.958988

**Published:** 2022-08-22

**Authors:** Leo Malandain, Konstantinos N. Fountoulakis, Timur Syunyakov, Evgeniia Malashonkova, Daria Smirnova, Florence Thibaut

**Affiliations:** ^1^Department of Psychiatry-Addictology, University Hospital Cochin (Hospital Tarnier, AP-HP), Paris, France; ^2^Third Department of Psychiatry, School of Medicine, Faculty of Health Sciences, Aristotle University of Thessaloniki, Thessaloniki, Greece; ^3^Education Center, Mental-health Clinic, N.A. Alexeev of Moscow Healthcare Department, Moscow, Russia; ^4^Zakusov Institute of Pharmacology, Moscow, Russia; ^5^International Centre for Education and Research in Neuropsychiatry, Samara State Medical University, Samara, Russia; ^6^Department of Psychiatry, Nord-Essonne Group Hospital, Orsay, France; ^7^Department of Psychiatry, Narcology, Psychotherapy and Clinical Psychology, Samara State Medical University, Samara, Russia; ^8^Institute of Personalized Psychiatry and Neurology, V.M. Bekhterev National Medical Research Center for Psychiatry and Neurology, Saint Petersburg, Russia; ^9^INSERM U1266, Institute of Psychiatry and Neuroscience, University of Paris Cité, Paris, France

**Keywords:** mental health, anxiety, depression, alcohol, tobacco, internet, COVID-19, lockdown

## Abstract

**Introduction:**

COVID-19 has enormous impacts on each individual. The goals of our study were (1) to assess the rate of internet and psychoactive substance use, clinical depression and anxiety in a French population during the lockdown (2) to study the role of clinical and socio-demographic variables (especially, gender).

**Materials and methods:**

During lockdown, an online anonymous questionnaire was used to assess socio-demographic and health data, previous psychiatric history, anterior and current internet and psychoactive substance use, current anxiety, depression and suicidal ideation. The associations of socio-demographic, clinical variables with anxiety, depression, internet or psychoactive substance use were examined.

**Results:**

The study included 263 participants (aged 38.1 ± 15.3−197 males and 64 females). During the lockdown, internet use increased in 14.4% of cases, alcohol use in 20.2%, and tobacco use in 6.8%. In contrast, more participants reported a decrease in alcohol, tobacco or illicit drug use (25.9, 24, and 27.8% respectively). Anxiety was reported in 62.4% and depression in 20.2% of cases; 29.7% of participants reported an increase in anxiety and 25.5% an increase in depression. Depression was associated with an increase in internet and tobacco use. Tobacco and alcohol use were positively associated and an increase in use was more frequent in previous users of both substances. Maintaining a daily routine and relationships with family, being self-employed were associated to lower risks of depression and anxiety.

**Conclusion:**

Higher rates of internet use, as well as depression and anxiety, were observed during the lockdown. Gender was not a significant associated factor.

## Introduction

The duration, spread and scalability of the COVID-19 pandemic as well as its health, economic and social impacts have had unprecedented consequences with tremendous impact on public mental health. The neurobiological and neuroinflammatory impacts of the virus, their consequences on the nervous system remain unclear (1, 2). Lifestyle changes, especially during the lockdown periods, with loss of social landmarks, changes in ways of working, daily habits, social rites (weddings, funerals, etc.), forced social isolation, have had psychological consequences that some authors have called a “psychiatric wave” (3). One meta-analysis identifies and includes 21 studies and 26 independent samples from Eastern Europe. The pooled prevalence of anxiety was 30% (95% CI: 24–37%) the pooled prevalence of depression was 27% (95% CI: 21–34%) (4). Salazar de Pablo et al. (5) in a meta-analysis, found that, on 60,458 individuals, there were predisposing factors for the development of psychiatric disorders, such as general health concerns, fear, poor sleep, or burnout for health professionals.

In the literature, the most frequent associated factors with an increase in anxiety and depression were: female gender (6–13) or non-binary gender (7, 14) and a young age (6, 8, 9, 11). Anxiety and depression are likely to trigger substance craving in women independently of other factors. However, mixed results were reported regarding a role of gender in psychoactive substance use changes. In fact, even if the fatality rate has been twice higher for men than for women, the COVID-19 pandemic has affected women more than men, both as frontline workers and at home (for review, see 14). Women are also more likely to have a lower socioeconomic status (e.g., lack of education, lower job status, worse financial situation, violent partners, history of sexual violence) which became even more frequent during the pandemic and may have worsened mental health status (15).

The goals of our study were (1) to assess the rate of psychoactive substance and internet use as well as clinical depression, anxiety and suicidal ideation in a French population during the lockdown; (2) to study the role of clinical and socio-demographic variables (especially, gender) in these changes.

## Materials and methods

### Methods

This study was part of a larger international study COMET-G, conducted in 40 countries including Greece and France. In France, the study took place during the lockdown period from 11 April 2020 to 1 May 2020. All data were collected online and anonymously. Participants were informed about this study through social networks and news sites. The study design was cross-sectional, the questionnaire was carried out once with estimation during the lockdown of alcohol and tobacco use as well as anxiety, depression, suicidal ideation and other addictive behaviors, in comparison with before lockdown. All participants gave their informed consent before completing the questionnaire. The study was approved by the Ethics Committee of the Faculty of Medicine of Thessaloniki, Greece. In France, the study was carried out in agreement with the Ethics Committees of the Nord-Essonne public hospital.

The protocol and questionnaire used in this study are shown in Supplementary Table 1. Demographic data and data pertaining to general health, previous psychiatric history, current symptoms of anxiety (using the STAI-Y1 state, 16), depression (using the CES-D, 17) and suicidal ideation (using the RASS, 18) were collected; as well as changes in sleep, tobacco and alcohol use, sexual behavior, family relationships, financial situation, eating and exercising and finally, religion/spirituality. Additionally, beliefs concerning the COVID-19 outbreak, including the measures taken and conspiracy theories, were investigated.

According to a previously developed method by Fountoulakis (18, 19), a cut-off score > 24 for the CES-D was used to identify clinical cases of major depression; a STAI-S score > 38 was used to identify clinical cases of anxiety. The cut-off score > 24 for CES-D and a derived algorithm were used to identify cases of probable depression. This algorithm used the weighted scores of selected CES-D items to arrive at the diagnosis of depression, and has already been validated (18, 19). Cases identified by both, the cut-off and the algorithm, were considered “depression.”

For alcohol and tobacco consumption, people who did not describe any change in their consumption were omitted, in order to study the factors linked to an increase in alcohol and tobacco use.

### Statistical analysis

A descriptive analysis was used for all data.

The associations of demographic, clinical variables with anxiety, depression or psychoactive substance (PAS) use were examined using univariate analyses. A Pearson’s chi-square test with *p* < 0.05 was used as criterion for the selection of variables to enter subsequent logistic regression analysis. During this step ordinal variables were treated as categorical. Then, selected variables for each dependent variable were entered into three stepwise forward logistic regression analyses to find the model that better describe anxiety, depression or PAS. Wald statistics were used to assess significance of predictors that were retained in the model. Parameter estimates and odds ratios with corresponding 95% confidence interval (CI) were estimated using unconditional logistic regression to understand a direction of link with dependent variables. The goodness-of-fit for the final model was tested using the misclassification rates. Statistical significance was defined as *p* < 0.05. Analyses were performed using TIBCO Statistica 13.3 software [*TIBCO Software Inc.* (20). *Statistica data analysis software system, version 13^1^* ].

## Results

### Population studied

The study sample included 263 participants (Table 1). There were no missing data. Among them, 2 declared themselves “*other gender than male or female*” (0.76%, aged 27.5 ± 10.6) and were included in the analysis.

**TABLE 1:** Socio-demographic description of the population.

	*N* = 263
**Gender**	
Male	79.4% (*n* = 197)
Female	24.3% (*n* = 64)
**Age (mean [SD])**	
Male	37.9 [15.5]
Female	39.0 [14.7]
Total	38.1 [15.3]
**Employment**	
Employed	46.8% (*n* = 123)
Self-employed	8.8% (*n* = 23)
Student	26.2% (*n* = 69)
Unemployed or retired	14.1% (*n* = 37)
Other*	4.2% (*n* = 11)
**Marital Status**	
Married or living with somebody	58.6% (*n* = 154)
Living alone	41.4% (*n* = 109)

*Other = No working by choice, disability pension and other sources of income.

### Descriptive epidemiological analysis

#### Before lockdown

A personal history of psychiatric disorders was reported as followed before the lockdown: 18.3% reported a depressive disorder (*n* = 48), 12.2% an anxiety disorder (*n* = 32), 0.4% a bipolar disorder (*n* = 1) and 2.7% another psychiatric disorder (*n* = 7). A history of suicide attempts was reported in 6.1% of cases (*n* = 16). Before lockdown, participants reported tobacco use in 20.5% of cases (*n* = 54); alcohol use was considered “too important” (“*more than one drink or its equivalent every day*”) in 12.6% of cases (*n* = 33); alcohol was used occasionally in 4.9% of participants (*n* = 13); and, finally, 1.1% reported frequent use of illicit drugs (*n* = 3) (defined by the answer “*often*” to the use of illicit substances). Psychotropic treatments were previously prescribed as follows: antidepressants in 4.6% of cases (*n* = 12), anxiolytics in 3.0% (*n* = 8). Three percent of participants (*n* = 8) received psychotherapy.

#### Mental health changes during the lockdown

Using STAI-S and CES-D, 29.7% (*n* = 78) of participants reported an increase in anxiety and 25.5% an increase in depressive symptoms (*n* = 67) during the lockdown as compared to before lockdown respectively. The mean (+/S.D.) scores of STAI-S, CES-D and RASS total score were 42.7 (SD = 12.7), 17.7 (SD = 6.6) and 9.0 (SD = 3.3) during the lockdown respectively. Using previously defined thresholds for STAI-S (>38) and CES-D (>24), clinical cases of anxiety during the lockdown were reported in 62.4% of participants (*n* = 164), and clinical cases of depression in 20.2% (*n* = 53). Suicidal thoughts were increased in 13.3% (*n* = 35) and decreased in 8.0% (*n* = 21) of cases as compared to before the outbreak.

Compared to before the outbreak, the participants reported a decrease in tobacco use in 24.0% of cases (*n* = 63) and an increase in 6.8% of cases (*n* = 18). A decrease in alcohol use was reported in 25.9% of cases (*n* = 68) and an increase in 20.2% (*n* = 53). A decrease in illicit drug use (such as cannabis) was observed in 27.8% of cases (*n* = 73) and an increase in 1.1% (*n* = 3).

An increase in social networks use [52.5% (*n* = 138)], and an increase in cybersex and/or gambling use were observed in 14.4% of cases (*n* = 38) as compared to before the lockdown.

Table 2 summarizes the impact of the COVID-19 lockdown on mental health.

**TABLE 2:** Impact of the lockdown on mental health (percentages of answers related to mental health).

		*n*	%
F21. How much has your emotional state changed in relation to the appearance of anxiety and insecurity compared to before the COVID-19 epidemic?	Much worse	65	24.7%
	Worse	13	4.9%
	It has not changed	158	60.1%
	A little bit better	18	6.8%
	Much better	9	3.4%
G21. How much has your emotional state related to the experience of joy or melancholy changed in comparison to before the COVID-19 epidemic?	It got a lot worse	55	20.9%
	It got a little worse	12	4.6%
	Neither better nor worse	160	60.8%
	Improved a bit	28	10.6%
	It has improved a lot	8	3.0%
O11. How much has your tendency to think about death and/or suicide changed, compared to before the outbreak of COVID-19?	Very much decreased	15	5.7%
	Decreased a bit	6	2.3%
	Neither increased, nor decreased	207	78.7%
	Increased a bit	32	12.2%
	Very much increased	3	1.1%
**Depression** (using a cut-off score ≥ 24 using the CES-D)	No	210	79.8%
	Yes (24 +)	53	20.2%
**Anxiety** (using a cut-off score ≥ 38 using the STAI-S)	No	99	37.6%
	Yes (38+)	164	62.4%

### Statistical analyses of the changes reported in mental health

We have studied the impact of the severe March 2020 lockdown in France on mental health in general and in particular on anxiety, depression as well as alcohol, tobacco and internet use.

The results of the univariate analysis appear in Supplementary Table 2. Based on χ^2^ square test’s *P*-value < 0.05 univariate analysis revealed variables as possible predictors. We have used the significantly associated variables for binary logistic regression analysis. The results of the binary logistic regression for depression, anxiety, alcohol or tobacco use and changes in internet use are shown in Table 3.

**TABLE 3:** Factors associated with clinical depression and anxiety during lockdown.

Effect°	S.E.	Wald	Odds Ratio	Lower CL 95%	Upper CL 95%	*p*
**DEPRESSION**						
L4°	0.250	16.418	0.363	0.222	0.593	<0.001**
G21°	0.205	9.366	1.870	1.252	2.792	0.002**
N4°	0.236	17.503	2.683	1.690	4.261	<0.001**
E2°	0.274	6.367	0.501	0.292	0.857	0.012*
K5°	0.532	4.948	0.306	0.108	0.869	0.026*
Anxiety	0.284	5.033	0.280	0.092	0.851	0.025*
B1°	0.227	3.521	1.532	0.981	2.391	0.061
**ANXIETY**						
F21°	0.188	16.661	2.157	1.491	3.121	<0.001**
C4°	0.214	19.432	0.389	0.256	0.592	<0.001**
Depression	0.277	4.732	3.340	1.127	9.899	0.030*
K1°	0.132	8.364	0.683	0.527	0.884	0.004**
B1°	0.194	7.219	1.685	1.152	2.464	0.007**
L1°	0.201	5.459	1.601	1.079	2.375	0.019*
M5°	0.242	5.014	0.581	0.361	0.935	0.025*

*p < 0.05; **p < 0.01.

Effect°:

**DEPRESSION**:

L4. I am having dreams in which I feel trapped, over the last 3 weeks.

G21. How much has your emotional state related to the experience of joy or melancholy changed in comparison to before the COVID-19 epidemic?

N4. Do you think sex helps you deal with your daily stress and anxiety?

E2. Do you want to receive emotional support from other members of your family during this period?

K5. Have you acquired internet-related habits that you did not have before (for example: created a Facebook account, engaging in cybersex or gambling)?

B1. In general, your health over the last month can be described as: excellent/very good/good/moderate/bad.

**ANXIETY**:

F21. How much has your emotional state changed in relation to the appearance of anxiety and insecurity compared to before the COVID-19 epidemic?

C4. Are you afraid that in case you contract the coronavirus, some people will step away from your life and behave to you in a different way later?

K1. The information and use of the internet worry me about the issue regarding the COVID-19?

B1. In general, your health over the last month can be described as: excellent/very good/good/moderate/bad.

L1. The quality of my sleep has changed recently. It is: much worse/a little bit worse/the same/a little better/much better.

M5. During lockdown, you drink alcohol compared to before: more than before/the same as before/less than before.

#### Clinical depression

Gender was not associated with clinical depression (*p* = 0.70).

The results of the binary logistic regression for depression are shown in Table 3. The results show that, apart from the general appreciation of the state of health (B1), some variables were significantly associated with clinical depression. For L4, E2, K5 and for anxiety, the higher the response scores, the greater the risk of depression. Conversely for variables G21 and N4, a higher score was associated with a lower risk of depression.

#### Clinical anxiety

Gender was not associated with clinical anxiety (*p* = 0.09).

The results of the binary logistic regression for clinical anxiety are shown in Table 3. The higher the scores for variables F21, B1, L1 and for depression, the lower the risk of anxiety, and the higher the scores for variables C4, K1, and M5, the higher the risk of anxiety.

#### Alcohol consumption

Gender was not associated with increasing alcohol consumption (*p* = 0.80).

The results of the binary logistic regression for alcohol consumption are shown in Table 4. The higher scores for variables M2 and M4 the higher probability of alcohol consumption increase (for M2: high score = “*I drank a lot before pandemic*,” for M4: high score = “*Smoke more than before*”).

**TABLE 4:** Factors associated with alcohol, tobacco consumption and change in internet use during lockdown.

Effect°	S.E.	Wald	Odds Ratio	Lower CL 95%	Upper CL 95%	*p*
**ALCOHOL**						
M4°	0.414	14.251	0.210	0.093	0.472	<0.001**
M2°	0.826	10.718	0.067	0.013	0.338	0.001**
**TOBACCO**						
M1°	0.938	8.087	0.069	0.011	0.437	0.004**
M6°	0.810	8.921	0.089	0.018	0.435	0.003**
M5°	0.586	6.601	0.222	0.070	0.700	0.010**
Depression	0.493	5.893	10.973	1.586	75.899	0.015*
**INTERNET CHANGE**						
K4°	0.435	11.951	4.493	1.917	10.530	<0.001**
C4°	0.159	6.859	1.517	1.111	2.073	0.009*
E3°	0.196	6.845	0.599	0.408	0.879	0.009*
O13°	0.396	3.809	2.166	0.997	4.705	0.0.51

*p < 0.05; **p < 0.01.

Effect°:

**ALCOHOL CONSUMPTION**:

M4. During lockdown, you smoke compared to before: more than before/the same as before/less than before.

M2. Alcohol use before the epidemic: more than before/the same as before/less than before.

**TOBACCO CONSUMPTION**:

M1. Smoking before the epidemic: more than before/the same as before/less than before.

M6. While isolated at home, you use illegal substances compared to before: more than before/the same as before/less than before.

M5. During lockdown, you drink alcohol compared to before: more than before/the same as before/less than before.

**CHANGE IN INTERNET USE**:

K4. How much do you use the social media while in isolation at home? more than before/the same as before/less than before.

C4. Are you afraid that in case you contract the coronavirus, some people will step away from your life and behave to you in a different way later? never/a little/moderately/much/very much.

E3. Are there any conflicts with the rest of your family members during this period? much less/less/same/more/much more.

O13. Have you ever attempted suicide, during your whole life so far? never/once/2-3 times/many times.

#### Tobacco consumption

Gender was not associated with increasing tobacco consumption (*p* = 0.32).

The results of the binary logistic regression for tobacco use are shown in Table 4. The higher the M1, M6 and M5 scores, the higher the risk of consuming tobacco and the higher depression was the higher the risk of increasing smoking (for M1: high score = “*I was smoking before pandemic*,” for M5: high score = “*Drink more than before*,” for M6: high score = « *Use illegal substances more than before*»).

#### Change in internet use

Change in internet use was defined by creating a new social media account, beginning online gambling or a cybersex activity.

Gender was not associated with an increase in internet use (*p* = 0.84). The results of the binary logistic regression for internet use are shown in Table 4.

Changes in use of internet were significatively associated with conflicts with family and the fear to be abandoned in case of contracting COVID-19. The increasing use of social networks during the lockdown was also associated with these changes.

## Discussion

The lockdown associated to the COVID-19 epidemic has had significant consequences on mental health in France. According to the thresholds we have chosen, 62.4% of the participants reported an anxiety disorder, 20.2% a depressive disorder and finally 13.3% of participants reported an increase in suicidal thoughts. Considering psychoactive substance use, a decrease in consumption was observed more frequently than an increase: an increase in alcohol consumption was reported in 20.2% of the population (a decrease in 25.9%), an increase in tobacco use in 6.8% (a decrease in 24.0%) and an increase in illicit substances in 1.1% (a decrease was observed in 27.8% of cases). Behavioral changes with an increase in Internet use (creation of new social network accounts, gambling, cybersex, etc.) were reported in 14.4% of participants. In our study, surprisingly, gender did not have any significant role in substance use changes, anxiety, depression or suicidal ideation. Additionally, in both sexes, an increase in the amount of food intake, a worsening in sleep (but only 3% were taking hypnotics), a decrease in physical exercise and inadequate frequency of sexual intercourses were reported in one third of participants, while 2/3 have maintained a basic daily routine during lockdown. Only half of participants reported excellent or very good health during the preceding month; half continued to work during lockdown. Interestingly 3/4 of participants found information on internet misinforming and/or misleading.

A recent study using the same methodology was conducted in a Greek population, during the COVID-19 pandemic (14). This study was carried out in 3399 Greek adults (621 males and 2757 females) during the lockdown. Clinical depression was observed in 9.3% of cases, severe distress in 8.5%, increased anxiety in 45.0% of participants and suicidal thoughts increased in 10.4%. Unfortunately, the rate of psychoactive substance use was not reported. As compared to Greece, we have found a higher prevalence of anxiety, depression and suicidal ideation. Gender effect was not analyzed in the Greek study. In addition, many previous studies have reported an increase in anxiety and depressive symptoms during the lockdown. A meta-analysis, including 31 studies (5153 adults), found a prevalence of pooled depression of 45.0%, pooled anxiety of 47.0% and pooled sleep disturbances of 34.0% respectively (21). Gender did not appear to be an associated factor or risk factor for the development of anxiety, depression or insomnia during the lockdown. A recent study conducted in 204 countries (48 studies included), reported a marked increase in the prevalence of major depressive disorders (27.6%) and anxiety disorders (25.6%) (22). Females were more affected by the pandemic than males. Another systematic review found an increase in symptoms of anxiety (in 6.33% to 50.9% of cases), depression (in 14.6% to 48.3%), post-traumatic stress disorder (in 7% to 53.8%), psychological distress (in 34.43% to 38%), and stress (in 8.1% to 81.9%) in the general population (countries: China, Spain, Italy, Iran, United States, Turkey, Nepal, and Denmark) (6). Female gender was associated with a greater risk of psychiatric symptoms (such as anxiety or depression) during the lockdown. Finally, in a French study including 69,054 students (72.8% of females), the prevalence rates were lower than those observed in our study with 11.4% of self-reported suicidal thoughts, 22.4% of severe distress (IES-R scale), 24.7% of perceived stress (PSS-10 scale), 16.1% of depression (BDI-13 scale), and 27.5% of anxiety (using STAI-Y2 scale) (7) during lockdown respectively. Female gender and non-binary gender were identified as a risk factor for depression or anxiety. In several European studies conducted during the lockdown, female gender was associated, with greater psychological distress or an increase in the level of stress (23–26). In contrast, Schmits (27) reported an increase in anxiety and depression (*N* = 2871 adults, 79.0% females) but without any gender effect. Finally, three studies were in line with these results by showing an increase in substance consumption and psychological distress but without any gender effect (28–30).

Several authors described changes in psychoactive substance use and addictive behaviors. Alcohol use disorder was one of the most frequent disorders observed during the lockdown (31, 32). For example, 5931 individuals completed the Alcohol Use Disorders Identification Test (AUDIT scale) in the United States (33). The prevalence of cases with AUDIT ≥ 15 increased from 7.9% to 29.1% during the severe lockdown, while the prevalence of cases with AUDIT ≥ 20 increased from 3.9% to 17.4%. In contrast, Kilian (34) found that about half of the respondents (11,295 adults from 18 Eastern European countries) indicating past-year alcohol or tobacco use reported no change in their consumption. In addition, among alcohol users who reported changes in their alcohol use, a larger proportion reported a decrease rather than an increase in most countries, the opposite was true for tobacco use; women, young adults and past-year harmful alcohol users were identified as being more likely to change their consumption. Similarly, in our population more participants reported a decrease rather than an increase in PSA use during the lockdown but without any gender effect. Daigre (35) found that out of 588 patients (29.3% of females), males reported significantly more frequent alcohol and cocaine consumption during lockdown, while females experienced more anxiety and depressive symptoms. In an Australian study including 13,739 individuals (54% of females), using the 8-item AUDIT test, the authors found that younger drinkers, particularly young women, decreased their consumption the most; in contrast, there was a small increase in consumption in middle-aged women (36). Barbosa (37) (*N* = 993 patients (52.3% of females), compared to before lockdown), reported that participants consumed more drinks per day (+29%, *p* < 0.001), and a greater proportion reported exceeding recommended drinking limits (+20%, *p* < 0.001) as well as binge drinking (+21%, *P* = 0.001). The percentage exceeding drinking limits was higher in women than in men (*P* = 0.026). Rodriguez (*N* = 754; 50% women) using Quantity/Frequency/Peak Alcohol Use Index (QF) found that COVID-19 psychological distress was associated with greater alcohol use in females but not in males (38). Currie (*N* = 933) found that more females (19%) than males (13%) met criteria for high pandemic-related PTSD symptomology, while a similar percentage (13.4% of females, 13.2% of males) reported a significant increase in substance use during the pandemic (39). High pandemic-related PTSD symptomology was associated with a significant substance use increase among both females (OR = 2.2) and males (OR = 2.3) in adjusted models. Jackson (*N* = 36,980 adults, 51.0% females) found a significant increase in smoking prevalence, with no gender effect, but a significant increase in high-risk drinking prevalence, with particularly high increases among women (40). Mougharbel (41) (*N* = 1,005, 49.6% females) found that females had higher odds of increased drinking and anxiety during lockdown. Thompson (2000 adults, 51.9% females) found that while females reported higher rates of emotional distress, significant associations with increased drinking frequency were only observed among males in gender-stratified analyses (42). On the contrary, Mongeau-Pérusse (43) found an increase in alcohol use in 847 participants (77.8% of females).

Among behavioral addictions, as observed in our study, internet addiction (particularly the use of social medias), cybersex, video gaming and online gambling, problematic internet use and problematic pornography use have shown a significant rise during lockdown (44) with an increase in connections to porn sites from 4 to 24% (45–47). Stress, boredom and isolation caused by confinement, may have played a significant role in this increase (48). In France, Attanasi (49) (*N* = 1,087, 74.7% of females) found that females were about 1.6 times more likely than males to lose control of their usual diet and about 2.3 times more likely than males to increase smartphone usage, while no significant gender effect was detected for increased videogame use. Grubbs (*N* = 868 participants, 21% of females) reported that pornography use tended downward over the pandemic in both men and women, and problematic pornography use trended downward in men and remained low and unchanged in women (50). These results suggested that fears about problematic pornography use during pandemic-related lockdowns were not fully supported by available data. In a recent meta-analysis, Marciano found that although most studies reported a positive association between ill-being and social media use (*r* = 0.171, *p* = 0.011) and ill-being and media addiction (*r* = 0.434, *p* = 0.024), with social comparison, fear of missing out, and exposure to negative contents as risk factors (51). Moreover, COVID-19 anxiety was related to both gambling problems (IRR: 1.05; 95% CI: 1.01–1.09) and online gaming problems (IRR: 1.03; 95% CI: 1.01–1.05) (52). Faced with this problematic use of the internet, some authors have proposed strategies not only to limit these disorders but also their impacts in terms of mental health, in particular with complications of depression and anxiety, such as general prevention with internet use embedded in daily routines and general lifestyles, and increasing contacts with relatives (53).

In our study, similarly to Fountoulakis et al. (54), many factors were associated with anxiety or depressive symptoms during this period. We found an association between the fact of being confined to a high level and anxiety as well as depression. Moreover, a previous prescription of anxiolytics increased the risk for suicidal ideation. On the other hand, maintaining a basic daily routine and relationships with family members were associated with lower risks of anxiety or depression. In addition, being self-employed was also associated to lower risks of depression and anxiety. Working in the medical field did not appear to have a protective or aggravating impact on mental health during the lockdown and continuing to work was not statistically associated with any change in mental health status.

An interesting significant association was observed between depression and behavioral changes in Internet use (creation of new social network accounts, gambling, cybersex, etc.). During the lockdown there was also a relationship between alcohol use, increase in tobacco use and previous alcohol use. We did not find any significant association between anxiety or depression and alcohol consumption. Finally, there was a positive relationship between tobacco use during the lockdown and previous smoking status, an increase in alcohol and other psychoactive substances use or with depression scores.

In the literature, in addition, not living with family members, having a low quality of social relations were associated with an impact on depression and anxiety (5, 9–11, 26). A lack of physical exercise, a low socio-economic level and unemployment were also associated to a worse outcome on mental health (6, 7, 11), as well as a large number of days of confinement (11). The presence of a psychiatric history was also strongly associated with the risk of relapse of mental illnesses during this period (3, 6, 10).

### Strengths and limitations of our study

The strength of this study was the prevalence of clinical depression, clinical anxiety and the increase observed in internet use during the lockdown period in France, as well as the factors associated with them, particularly the profound socio-professional changes that this crisis has generated. The major limitation of the study were that a lower number of women were included, which may have contributed to the lack of gender effect. The relatively small sample size of French participants and the use of threshold scores from a larger population in Greece may also be a measurement bias. The study sample was too small to weight data for its representativeness in relation to French general population but despite a small sample size French sample data were similar to European countries’ rates in the COMET-G projects countries of comparison which let us recognize the findings as reliable to make the conclusions.

Finally, the different activities grouped under the term “changes in internet use” could not be differentiated, and it will be interesting to be able to dissociate these different uses in future research to assess the links with mental health during the containment of COVID-19.

## Conclusion

Our study reported higher rates of internet use (creation of new social network accounts, gambling, cybersex, etc.), as well as depression, anxiety and suicidal thoughts in the general population during the lockdown in France. More participants reported a decrease than an increase in alcohol, tobacco or illicit drug use. Surprisingly, gender was not a significant associated factor. A high degree of confinement was associated with depression and anxiety. Depression was associated with an increase in tobacco and internet use. Tobacco and alcohol use were positively associated and an increase in use was more frequent in previous users of both substances. In contrast a previous psychiatric history was not associated to any change. Finally, maintaining a basic daily routine and relationships with family members, being self-employed were associated with lower risks of depression and anxiety.

This worldwide psychiatric wave secondary to COVID-19 pandemic is a major health concern with medium and long-term consequences, which must be sought, prevented and treated.

## Data availability statement

The original contributions presented in this study are included in the article/Supplementary material, further inquiries can be directed to the corresponding author.

## Ethics statement

The studies involving human participants were reviewed and approved by the Ethics Committee of the Faculty of Medicine of Thessaloniki, Greece. In France, the study was carried out in agreement with the Ethics Committees of the Nord-Essonne Public Hospital. The patients/participants provided their written informed consent to participate in this study.

## Author contributions

KF and DS designed the study. TS wrote the analysis plan and undertook the statistical analysis. EM participated in the inclusion of patients. LM and FT managed the literature searches and wrote the first draft of the manuscript. All authors contributed to and approved the final manuscript.

## Footnotes

1
https://tibco.com
